# Ambroxol as a novel disease-modifying treatment for Parkinson’s disease dementia: protocol for a single-centre, randomized, double-blind, placebo-controlled trial

**DOI:** 10.1186/s12883-019-1252-3

**Published:** 2019-02-09

**Authors:** C. R. A. Silveira, J. MacKinley, K. Coleman, Z. Li, E. Finger, R. Bartha, S. A. Morrow, J. Wells, M. Borrie, R. G. Tirona, C. A. Rupar, G. Zou, R. A. Hegele, D. Mahuran, P. MacDonald, M. E. Jenkins, M. Jog, S. H. Pasternak

**Affiliations:** 1 Cognitive Neurology and Alzheimer’s Disease Research Centre, Parkwood Institute – Main Building, Room A230, 550, Wellington Road, London, Ontario N6G 0A7 Canada; 2 Lawson Health Research Institute, London, Ontario Canada; 3 Deparment of Clinical Neurological Science, Schulich School of Medicine and Dentistry, Western University, London, Ontario Canada; 4 Division of Geriatric Medicine, Schulich School of Medicine and Dentistry, Western University, London, Ontario Canada; 5 Department of Biochemistry, Schulich School of Medicine and Dentistry, Western University, London, Ontario Canada; 6 Department of Medicine, Schulich School of Medicine and Dentistry, Western University, London, Ontario Canada; 7 Department of Physiology and Pharmacology, Schulich School of Medicine and Dentistry, Western University, London, Ontario Canada; 8 Department of Medical Biophysics, Schulich School of Medicine and Dentistry, Western University, London, Ontario Canada; 9 Department of Epidemiology and Biostatistics, Schulich School of Medicine and Dentistry, Western University, London, Ontario Canada; 10 Laboratory of Medicine and Pathobiology, The Hospital for Sick Children, Toronto, Ontario Canada; 11 Robarts Research Institute, Western University, London, Ontario Canada

**Keywords:** Parkinson’s disease dementia, α-Synuclein, Glucocerebrosidase, Ambroxol, Cognition

## Abstract

**Background:**

Currently there are no disease-modifying treatments for Parkinson’s disease dementia (PDD), a condition linked to aggregation of the protein α-synuclein in subcortical and cortical brain areas. One of the leading genetic risk factors for Parkinson's disease is being a carrier in the gene for β-Glucocerebrosidase (GCase; gene name *GBA1*). Studies in cell culture and animal models have shown that raising the levels of GCase can decrease levels of α-synuclein. Ambroxol is a pharmacological chaperone for GCase and is able to raise the levels of GCase and could therefore be a disease-modifying treatment for PDD. The aims of this trial are to determine if Ambroxol is safe and well-tolerated by individuals with PDD and if Ambroxol affects cognitive, biochemical, and neuroimaging measures.

**Methods:**

This is a phase II, single-centre, double-blind, randomized placebo-controlled trial involving 75 individuals with mild to moderate PDD. Participants will be randomized into Ambroxol high-dose (1050 mg/day), low-dose (525 mg/day), or placebo treatment arms. Assessments will be undertaken at baseline, 6-months, and 12-months follow up times. Primary outcome measures will be the Alzheimer’s disease Assessment Scale-cognitive subscale (ADAS-Cog) and the ADCS Clinician’s Global Impression of Change (CGIC). Secondary measures will include the Parkinson’s disease Cognitive Rating Scale, Clinical Dementia Rating, Trail Making Test, Stroop Test, Unified Parkinson’s disease Rating Scale, Purdue Pegboard, Timed Up and Go, and gait kinematics. Markers of neurodegeneration will include MRI and CSF measures. Pharmacokinetics and pharmacodynamics of Ambroxol will be examined through plasma levels during dose titration phase and evaluation of GCase activity in lymphocytes.

**Discussion:**

If found effective and safe, Ambroxol will be one of the first disease-modifying treatments for PDD.

**Trial registration:**

ClinicalTrials.gov NCT02914366, 26 Sep 2016/retrospectively registered.

## Background

Parkinson’s disease dementia (PDD) is a highly prevalent evolution of Parkinson’s disease [1], believed to result from the aggregation of α-synuclein in subcortical and cortical neurons [2, 3]. Longitudinal studies have shown that by the 10-year follow up from diagnosis, 46% of individuals with Parkinson’s disease had progressed to PDD [4]. Importantly, decline in cognitive function contributes to decreased quality of life [5], increased risk of institutionalization [6] and mortality [7] in individuals with Parkinson’s disease. Therefore, it is not surprising that the development of new therapies to treat cognitive decline has been rated by individuals with Parkinson’s disease and caregivers as one of the top 10 priorities in Parkinson’s disease research [8].

Currently, the treatment for cognition in PDD includes the use of cholinesterase inhibitors in addition to dopaminergic medication [9]. Although somewhat effective in their role of alleviating symptoms, these therapies do not affect the underlying mechanisms leading to progression of the disease and patients get worse despite treatment. Thus, the development of disease-modifying treatments for PDD is critical.

A potential target for disease-modifying treatment is the enzyme β-Glucocerebrosidase (GCase; gene name *GBA1*). GCase is a degradative enzyme that resides in the lysosome and cleaves a neutral glycolipid, glucocerebrosidase, present in the membranes of most cells. Having mutations in both alleles of *GBA1* results in Gaucher disease, which can manifest with hepatic, splenic, hematological and bone abnormalities (Type 1) or with a devastating neurological deterioration that can be rapid or chronic (Type 2 and 3, respectively). However, being a carrier for a pathological mutation in *GBA1* is one of the strongest genetic risk factors for Parkinson’s disease, PDD and Lewy Body dementia. Parkinson’s disease patients bearing *GBA1* mutation, tend to have earlier onset, a greater prevalence of cognitive decline as well as more severe cognitive impairment compared to those without the mutation [10]. In addition, reductions in GCase activity may play a role even in sporadic Parkinson’s disease, as these individuals have low levels of GCase in the brain and cerebrospinal fluid [11–13].

Laboratory studies have demonstrated a direct link between GCase activity and α-synuclein accumulation. In cultured cells, loss of GCase results in α-synuclein accumulation and this process feeds back upon itself, with overexpression of α-synuclein further inhibiting GCase function, and increasing GCase expression reducing α-synuclein [14]. Moreover, reducing GCase genetically [15, 16] or pharmacologically [17] in animal studies results in increased α-synuclein aggregates. Remarkably, overexpressing GCase in the brain of a Parkinson’s disease mouse model reduces α-synuclein and improves cognition [15, 18]. Taken together, these findings suggest that increasing GCase levels could be a therapy that addresses the underlying pathophysiology of PDD to modify the course of disease progression.

Ambroxol is an expectorant that has been available for adults and children over the counter in more than 50 countries for over 30 years [19]. Ambroxol was identified by the Mahuran Lab in a High Throughput Screen as a pharmacological chaperone of GCase, an agent that stabilizes and increases the levels of GCase [20]. Pharmacological chaperones are small molecules that bind to proteins to stabilize them, increasing the number of protein molecules that fold and function correctly, and thereby reduce the amount of protein degraded. They have been proposed as therapies for a wide range of inborn errors of metabolism, a large fraction of which are caused by mutations which destabilize protein folding, leading to degradation [21–23].

In addition, Ambroxol has good lipophilicity (cLogP = 2.8) and low polar surface area (PSA 58 Å^2^), predicting good CNS penetration. Ambroxol has an excellent safety record and has been studied in > 15,000 patients in more than 100 trials. While the normal expectorant dose in adults is in the range of 75–100 mg/day, doses of 1000 mg IV are used in pregnant women experiencing premature delivery to aid fetal lung maturation, and doses of 30 mg/kg in neonates for fetal respiratory distress syndrome [19].

Although Ambroxol was originally identified as a drug which stabilizes GCase bearing disease-causing mutations which increase its degradation, it is also able to increase the levels of normal GCase [20, 24]. As such, it can markedly increase GCase protein and activity in both normal and Gaucher disease fibroblasts (bearing GCase mutations) at concentrations in the micromolar range [20, 24]. Ambroxol treatment has also been shown to improve lysosomal biochemistry overall by indirect activation of a lysosomal master regulatory gene/transcription factor called TFEB and improved clearance of α-synuclein [25]. More recently, Ambroxol was found to raise GCase levels and lower α-synuclein in induced pluripotent stem cell (iPSC)-derived dopaminergic neurons from *GBA1* mutation patients [26].

In mice, oral Ambroxol for 12 days increased GCase activity in the brainstem, midbrain, cortex, and striatum of wild-type as well as transgenic mice carrying a L444P mutation (i.e. a *GBA1* mutation) [27]. The same study found that transgenic mice overexpressing human α-synuclein treated with Ambroxol had a reduction in α-synuclein levels in the brainstem and striatum compared to untreated mice. Furthermore, Ambroxol reduced S129 phosphorylation of α-synuclein (suggested to play a critical role in α-synuclein aggregation and formation of Lewy bodies and neuritis) in the brainstem by 41% in treated mice compared to untreated mice. Ambroxol has also recently been demonstrated to increase GCase levels in non-human primates [28]. Together, these results support the potential for using Ambroxol as a disease-modifying treatment for synucleinopathies such as PDD.

In pilot studies in humans, Ambroxol was effective at improving GCase function [29, 30]. In a trial aimed at Type 1 (non-neurological) Gaucher disease, 12 patients received 150 mg/day for 6 months, and all but one had some measurable improvement. The best response was in the lightest patient (who received 3 mg/kg/day), suggesting that Ambroxol was under dosed in this study. Ambroxol has also been administered to five Japanese Gaucher disease patients with severe neurological disease, at 25 mg/kg/day or a maximum dose of 1300 mg/day for 6–48 months [29]. Despite their advanced disease, patients had improvements in neurological symptoms (decreased myoclonus and seizure frequency). Improvements in myoclonus allowed two patients to stand steadily, control their balance, and walk again. To our knowledge, no study has tested the effects of Ambroxol on people with Parkinson’s disease or PDD.

The main objectives of the present randomized controlled trial are 1) to demonstrate the safety and tolerability of Ambroxol in patients with PDD, 2) to examine the effects of Ambroxol on cognitive, biochemical, and neuroimaging measures, and 3) to acquire additional pharmacokinetic and pharmacodynamics data for use in future trials. It is hypothesized that Ambroxol will be safe and well tolerated in PDD, and may improve the course of cognitive impairment or motor function, or biomarkers of neurodegeneration in PDD by raising GCase levels in blood and CSF.

## Methods

The study has been approved by Western University Health Science Research Ethics Board (Protocol ID: 105234) and Health Canada (Protocol ID: HC6–24-c181033) in November 2015. The trial has been registered on ClinicalTrials.gov (ID: NCT02914366).

### Design

**Fig. 1:**
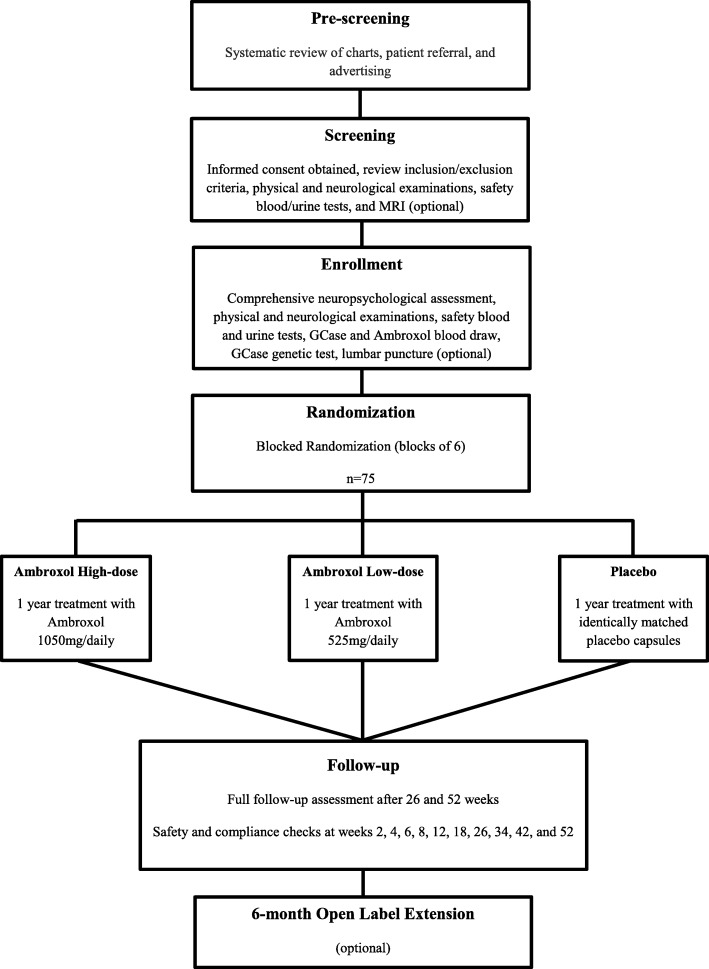
Trial flow diagram

This is a phase II, single-centre, double-blind, placebo-controlled randomized clinical trial (RCT) using a parallel arm design with a 6-month open label extension period (Fig. 1).

### Participants

Participants must fulfill the following inclusion criteria: 1) age greater than 50 years old; 2) Parkinson’s disease (Hoehn & Yahr stage 2–3.5) clearly established for more than 1 year before the onset of dementia; 3) mild to moderate dementia (defined by an upper cut off of a Montreal Cognitive Assessment score of 24 or below and the lower bound by a Mini Mental State Exam of 16 or greater); 4) be on stable doses of medications for motor (i.e. levodopa, dopaminergic agonist), cognitive (i.e. cholinesterase inhibitor), and psychiatric (i.e. antidepressant, antipsychotic) symptoms 3 months prior to the study; and 5) have a responsible caregiver at least 4 days/week. Potential participants will be excluded if they meet one of the following exclusion criteria: 1) have a clinically significant stroke or other neurological condition; 2) have other serious health underlying condition (e.g. cancer or unstable cardiac disease etc.).

Potential participants will be recruited from the Movement Disorders Clinics and Movement Disorders Centre at the London Health Sciences Centre, the Cognitive Neurology and Alzheimer’s disease Research Centre and the Aging Brain and Memory Clinic both at Parkwood Institute (all located in London, ON, Canada), and from community Neurologists that routinely manage Parkinson’s disease. Trial advertisement will be posted on strategic locations, shared in support groups, website, and social media via the Parkinson’s Society South-Western Ontario as well as the online platform Fox Trials Finder. Individuals referred to the study by physicians and those who spontaneously contact the research team will be pre-screened via chart inspection and, if meet inclusion criteria, be offered a Screening visit.

All study visits will take place at Parkwood Institute. MRI scans will be performed at Robarts Research Institute and gait assessment at the Movement Disorders Centre at London Health Sciences Centre all located in London, ON, Canada.

### Screening visit

In the screening visit, the inclusion and exclusion criteria will be reviewed followed by the performance of the Mini-Mental State Exam (MMSE) [31] and the Montreal Cognitive Assessment (MoCA) [32]. Then, participants’ demographic information, medical history, and concomitant medication list will be recorded. A neurological and physical examination will be performed by a Neurologist (SHP). On a separate day, participants will have an MRI scan to rule out possible confounding neurological disease such as stroke or brain tumor and as a baseline for MRI-based biomarkers studies. Individuals with contra-indications to MRI or who choose not to have a MRI scan will have a CT scan. Individuals who pass the Screening visit will complete the Baseline assessment within 28 days.

### Randomization and blinding

Randomization procedure will be completed by pharmacy staff using the website Research Randomizer (https://www.randomizer.org). Eligible participants will be randomly assigned into one of three arms: Ambroxol 1050 mg/day (High-dose), Ambroxol 525 mg/day (Low-dose) or placebo, in a 1:1:1 ratio in blocks of six participants each at baseline. Participants and all research personnel, with the exception of pharmacy staff, will be blinded to the randomization until the study is completed or a patient is purposely unblinded.

For blinding purposes, participants in all three arms will receive the same number of capsules per day. Medication will be blinded by over-encapsulating Ambroxol 75 mg capsules (commercial product) and a matching placebo compound (microcrystalline cellulose) using opaque gelatin capsules (blue colour). Medications are packed as individual patient quantities consisting of one blister card for each week of treatment. The investigational product will be labeled by pharmacy in a double-blind manner (Ambroxol 75 mg/placebo), with pre-assigned participant identification number.

### Intervention

The treatment regimen consists of a titration phase and a maintenance phase. Participants begin the titration phase (weeks 1 and 2) taking 3 capsules a day (225 mg or 150 mg or 0 mg) divided BID (2 capsules in the morning and 1 capsule in the evening). The first medication dose will be taken at the end of Baseline visit and in the presence of the study Neurologist. Possible side effects will be observed for 30 min. Medication dose will increase every two weeks to 6 capsules on weeks 3 and 4, 10 capsules on weeks 5 and 6, and 14 capsules on weeks 7 and 8 divided BID. At the end of titration (week 8) participants will have reached a maximum of 1050 mg or 525 mg or 0 mg per day, depending on group allocation. In the maintenance phase, participants will remain in their maximum dose (1050 mg or 525 mg or 0 mg) from week 9 to week 52 (End of Trial).

### Assessment procedures

Participants will complete 11 visits to Parkwood Institute including baseline, weeks 2, 4, 6, 8, 12, 18, 26, 34, 42, and 52 within one year of treatment. A comprehensive neuropsychological battery will be performed at baseline, week 26, and week 52. The assessment of participants’ motor and neuropsychiatric symptoms will also occur at these time-points. Physical examination will occur at all time-points but weeks 2 and 8, whereas neurological examination will occur at baseline, weeks 6, 12, 18, 34, 42, and 52. Blood and urine samples as well as electrocardiograms will be collected at all visits. Cerebrospinal fluid will be collected through lumbar puncture at baseline, weeks 12, and 52 (optional). Finally, MRI scans will be collected at Screening and week 52 (optional). Table 1 illustrates all assessments performed at each time-point for the duration of the trial.

**Table 1:** List of assessments performed in each visit

Visit Name	Screen	Baseline	Week 2	Week 4	Week 6	Week 8	Week 12	Week 18	Week 26	Week 34	Week 42	Week 52
Consent	X											
Medical History												
Medical History	X											
Demographics	X											
Review inclusion and exclusion criteria	X	X										
MRI	X											X
Randomization		X										
Medication												
In clinic first dose		X										
Compliance assessment			X	X	X	X	X	X	X	X	X	X
Concomitant Medication Check	X	X	X	X	X	X	X	X	X	X	X	X
Safety Assessments												
Adverse Event Review		X	X	X	X	X	X	X	X	X	X	X
Interview	X	X	X	X	X	X	X	X	X	X	X	X
Chemistry Blood Draw	X	X	X	X	X	X	X	X	X	X	X	X
Hematology Blood Draw	X	X	X	X	X	X	X	X	X	X	X	X
Coagulation Blood Draw	X	X	X	X	X	X	X	X	X	X	X	X
Genetics Blood Draw		X										
GCase Blood Draw		X	X	X	X	X	X		X			X
Ambroxol Blood Draw		X	X	X	X	X	X		X			X
Urinalysis	X	X	X	X	X	X	X	X	X	X	X	X
Standing Blood Pressure	X	X	X	X	X	X	X	X	X	X	X	X
Seating Blood Pressure	X	X	X	X	X	X	X	X	X	X	X	X
Height	X											
Weight	X	X	X	X	X	X	X	X	X	X	X	X
ECG	X	X	X	X	X	X	X	X	X	X	X	X
Physical Exam	X	X		X	X		X	X	X	X	X	X
Neurological Exam	X	X			X		X	X	X	X	X	X
CSF GCase		X					X					X
CSF Ambroxol		X					X					X
CSF Safety		X					X					X
MMSE	X	X		X	X		X	X	X	X	X	X
Gait Kinematics		X							X			X
Timed up and Go		X							X			X
Purdue Pegboard		X							X			X
UPDRS		X							X			X
Cognitive Assessments												
ADAS-Cog		X							X			X
CGIC		X							X			X
PD-CRS		X							X			X
NPI		X							X			X
CDR		X							X			X
MoCA		X							X			X
Trail Making Test		X							X			X
Stroop test		X							X			X
Geriatric Depression Scale		X							X			X

Legend: *MRI* Magnetic Resonance Imaging, *GCase* Glucocerebrosidase, *ECG* Electrocardiogram, *CSF* Cerebrospinal Fluid, *MMSE* Mini-Mental State Examination, *UPDRS* Unified Parkinson’s disease rating scale, *ADAS-Cog* Alzheimer’s disease Assessment Scale-cognitive subscale, *CGIC* ADCS–Clinician’s Global Impression of Change, *PD-CRS* Parkinson’s disease Cognitive Rating Scale, *NPI* Neuropsychiatric Inventory, *CDR* Clinical Dementia Rating Scale, *MoCA* Montreal Cognitive Assessment

### Primary outcome measures

The primary outcome measures will be the Alzheimer’s disease Assessment Scale-cognitive subscale (ADAS-Cog) [33] and the Alzheimer’s Disease Cooperative Study Clinician’s Global Impression of Change (ADCS-CGIC) [34]. The ADAS-Cog is a comprehensive assessment battery composed by 11 items (word-recall, commands, constructional praxis, naming, ideational praxis, orientation, word recognition, remembering instructions, comprehension, word finding, and spoken language). The test is rated on a scale from 0 to 70, where greater score represents greater impairment. Although designed for Alzheimer’s disease where it is considered a gold standard, the ADAS-Cog has been used effectively in many clinical trials of PDD including large randomized trials [33, 35–38].

The Alzheimer’s Disease Cooperative Study Clinician’s Global Impression of Change (ADCS-CGIC) scale [34] is a 7-point scale for rating patient function in cognition, behavior, and activities of daily living. ADCS-CGIC is a standard test in clinical trials in Alzheimer’s disease and has been useful in trials with PDD [37–39]. This scale was also included because the FDA requires measures of global function in cognitive clinical trials [40]. Thus, this measure will provide critical evidence for potential multi-centre trials in the future. At baseline, information regarding mental/cognitive state (arousal/attention, orientation, memory, language, praxis, and judgment/problem solving), behaviour (thought content, hallucinations/delusions, behaviour/mood, sleep and appetite, and neurological/psychomotor), and functioning (instrumental functional ability and social function) are collected from both patient and caregiver. This information will be used as a reference for the assessment of change throughout the study. In this clinical trial, the individual performing the ADCS-CIGC will be an independent rater blinded to participant’s progression in the trial (i.e. group allocation, compliance, adverse events, and performance in other outcome measures).

### Secondary outcome measures

#### Neuropsychological tests

Secondary cognitive measures include the Clinical Dementia Rating Scale [41], Parkinson’s disease Cognitive Rating Scale [42], Trail Making Test [43], and Stroop test [44].

#### Motor function

The assessment of motor function will be completed using the Movement Disorders Society Unified Parkinson’s Disease Rating Scale (MDS-UPDRS) [45], Purdue Pegboard [46], Timed Up and Go [47], and gait kinematics.

#### Mood and neuropsychiatric symptoms

Neuropsychiatric symptoms will be assessed with the Neuropsychiatric Inventory [48] and Geriatric Depression Scale [49].

#### CSF and brain imaging biomarkers

CSF collected through lumbar puncture (levels of α-synuclein, Tau, phospho-Tau and beta amyloid-42) and brain imaging (brain ventricle and hippocampal volume, and metabolic indicators of neurodegeneration) biomarkers will be collected.

#### Pharmacokinetics and pharmacodynamics

Pharmacokinetics and pharmacodynamics of Ambroxol will be examined through blood concentrations during a dose titration phase and measurements of GCase activity in lymphocytes.

#### Genetics

The *GBA1* gene will be sequenced in all participants to identify carriers and non-carriers of mutations and any other allelic variations in the gene for β-Glucocerebrosidase (GCase; gene name *GBA1*).

### Safety

Routine blood draws (chemistry, hematology, and coagulation) and urine chemistry will be collected in all visits, and any abnormal results will be shared with each participant’s family physician (primary care) with each participant’s permission. Results from laboratory analyses will be reviewed by the study’s principal investigator and periodically by an independent data and safety monitoring board (DSMB).

Each participant will be asked at every visit whether or not any adverse event occurred. Data on adverse events will be reviewed by the study’s principal investigator, recorded in participants’ files, and presented to the DSMB at meetings that occur every 6 months. Serious adverse events (death, hospitalization, or leading to disability/incapacity) will be reported to both Western University research ethics board as well as Health Canada.

Ambroxol is generally well tolerated [50]. The European Core Safety Profile lists headache, dizziness, tachycardia, flushing, nausea, vomiting, diarrhea and abdominal pain as side effects [51]. Recently severe skin lesions such as Stevens-Johnson Syndrome and toxic epidermal necrolysis (TEN) was added as a warning, but it appears to be so rare that it is not possible to determine an event rate. We were unable to identify any literature or case reports of severe reactions. A recent Post Marketing survey of 2707 individuals taking Ambroxol syrup found the 2.5% of patients reported adverse events, which were mostly mild, mainly GI (abdominal pain, diarrhea, nausea, vomiting). There were no serious adverse events.

In the event of minor adverse events (e.g. gastrointestinal side effects), patients will be allowed to spread their dose over a longer interval, or to reduce their dose to the highest dose tolerated. The dose can be advanced again after 2 weeks. For significant but non-threatening laboratory abnormalities, doses can be reduced to the highest dose tolerated. Participants will be allowed to stop taking medication if there is concern about a drug reaction of a clinically significant alteration in laboratory values. They can be re-challenged 2–4 weeks after these abnormalities have resolved.

In order to confirm participant self-reported adherence to the study medication, the number of pills returned in each visit will be counted and recorded. Unused pills will be returned to pharmacy for destruction.

### Sample size

Because Ambroxol has never been used for this indication, there is insufficient information available to perform a sample size calculation. However, it is known that individuals with Alzheimer’s disease typically have 6–7 points change in the ADAS-Cog in 1 year [52] and there are data suggesting that PDD patients may decline in a similar fashion [53–56]. The required sample size was calculated to ensure power of at least 80% in detecting two a priori comparisons, i.e., 1) the difference in average change in the ADAS-Cog scores between patients receiving Ambroxol and placebo, and 2) the difference in the ADAS-Cog scores between low dose Ambroxol and high dose Ambroxol, using 2-sided tests at the 5% significance level. The calculation assumed that the one-year change scores from baseline in ADAS-Cog scores are 7 points, 5 points, and 0 points for placebo, low dose, and high dose groups, respectively, with a common standard deviation of 5.5 points. Using the procedure ‘SAS proc glmpower’ (from SAS version 9.3, SAS Institute Inc., Cary, NC, USA), a total of 60 patients (20 per group) will provide 83.6% power to detect a difference of 2.5 between the average of two Ambroxol groups and placebo, and 80.7% power to a difference of 5 points between the low and high doses of Ambroxol. To account for attrition, 5 additional patients per group will be enrolled in the study.

### Statistical analysis

Descriptive statistics such as mean, standard deviation, and frequency by treatment arm will be used to summarize patient baseline characteristics.

The ADAS-Cog data will be analyzed using a restricted maximum likelihood-based repeated measures approach. The analyses will include the fixed, categorical effects of treatment, visit time, and the continuous, fixed covariate of baseline ADAS-Cog score, and interaction between treatment and visit. An unstructured (co)variance structure shared across treatment groups will be used to model within-patient errors. The Kenward-Roger approximation will be used to estimate denominator degrees of freedom and adjust standard errors. The primary analysis will be the contrast at week 52, comparing i) the average of the two Ambroxol groups versus placebo; ii) low-dose Ambroxol group versus high-dose Ambroxol group. All tests will be carried out at 2-sided 5% significance level.

Similar approach will be adopted for ADCS-CGIC and the secondary outcomes that are continuous. Binary outcomes will be analyzed using logistic regression analysis.

All analyses will be done using SAS version 9.3 (SAS Institute Inc., Cary, NC, USA).

## Discussion

Cognitive decline and its implications to the life of individuals with PDD represent a significant burden to patients, caregivers, and the health care system. Current pharmacological therapies are helpful for cognitive symptoms in PDD but are unable to modify the course of disease progression, making treatment for cognition in PDD a major unmet need. Increasing levels of the GCase enzyme has been proposed as a potential therapy for slowing neurodegeneration in Parkinson’s disease, and consequently PDD. There is increasing evidence in cell culture and animal models of PD that Ambroxol can raise levels of GCase. Strikingly, increased levels of GCase were accompanied by decreased levels of α-synuclein aggregation in brain areas of mice treated with Ambroxol. The present trial is the first to test Ambroxol in human subjects with PDD. The effects of Ambroxol will be measured using standardized cognitive, functional, behavioural, biochemical, as well as brain imaging outcomes. Results from this clinical trial may have a large impact in the research of disease-modifying therapies for PDD and other synucleinopathies including Parkinson’s disease and Lewy Body dementia. Importantly, Ambroxol is a drug already commercially available in most of the world and has an excellent safety record. Thus, repurposing Ambroxol for the treatment of PDD may expedite a potential disease-modifying treatment and circumvent a lengthy drug development process.

## Abbreviations

ADAS-Cog: Alzheimer’s disease assessment scale-cognitive subscale
ADCS-CGIC: Alzheimer’s disease cooperative study clinician’s global impression of change
CSF: Cerebrospinal fluid
DSMB: Data and safety monitoring board
*GBA1*: Gene encoding the protein β-Glucocerebrosidase
GCase: β-Glucocerebrosidase
iPSC: induced pluripotent stem cell
MMSE: Mini-mental state exam
MoCA: Montreal cognitive assessment
MRI: Magnetic resonance imaging
PDD: Parkinson’s disease dementia
TEN: toxic epidermal necrolysis
